# Sex ratios and gender norms: why both are needed to understand sexual conflict in humans

**DOI:** 10.1017/ehs.2024.3

**Published:** 2024-01-30

**Authors:** Renée V. Hagen, Brooke A. Scelza

**Affiliations:** Department of Anthropology, University of California, Los Angeles. United States of America

**Keywords:** gendered conflict, bargaining, sex ratio, gender ideology, gender norms

## Abstract

Sexual conflict theory has been successfully applied to predict how in non-human animal populations, sex ratios can lead to conflicting reproductive interests of females and males and affect their bargaining positions in resolving such conflicts of interests. Recently this theory has been extended to understand the resolution of sexual conflict in humans, but with mixed success. We argue that an underappreciation of the complex relationship between gender norms and sex ratios has hampered a successful understanding of sexual conflict in humans. In this paper, we review and expand upon existing theory to increase its applicability to humans, where gender norms regulate sex ratio effects on sexual conflict. Gender norms constrain who is on the marriage market and how they are valued, and may affect reproductive decision-making power. Gender norms can also directly affect sex ratios, and we hypothesize that they structure how individuals respond to market value gained or lost through biased sex ratios. Importantly, gender norms are in part a product of women's and men's sometimes conflicting reproductive interests, but these norms are also subject to other evolutionary processes. An integration of sexual conflict theory and cultural evolutionary theory is required to allow for a full understanding of sexual conflict in humans.

## Graphical abstract

**Figure:**
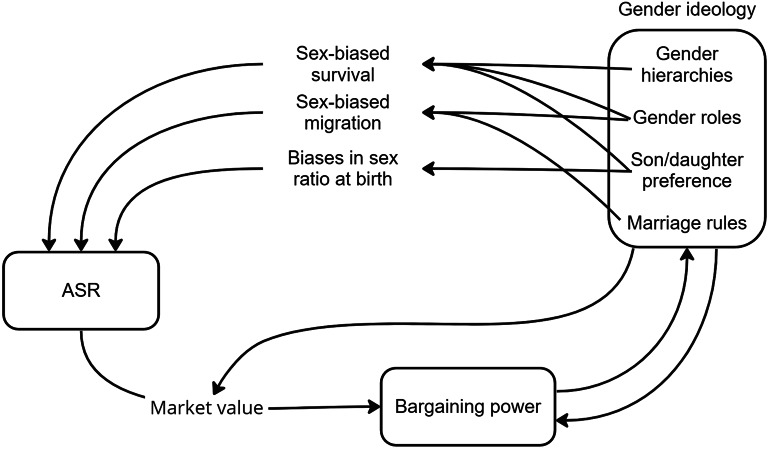

**Social media summary:** Hagen and Scelza argue for the integration of sexual conflict and cultural evolutionary theory to understand sexual conflict in humans.

## 1. Introduction

When two individuals mate, they have both converging and diverging interests. They share an interest in the success of any joint offspring, but may differ in the optimal trade-offs between current and future reproduction, or in the benefits they may be able to gain through inclusive fitness (Parker, 1979; Arnqvist & Rowe, 2005). Conflict occurs any time two partners cannot simultaneously reach their optimal fitness outcome (Parker, 2006). Sexual conflict theory is a framework in evolutionary biology that seeks to explain how such conflicts result in adaptations over evolutionary time, including behavioural flexibility within the lifespan of individuals. One important determinant of conflict is the number of reproductive options that each party has outside of their current partnership, which regulates how much bargaining power each partner has.

Anthropologists are increasingly applying sexual conflict theory to understand human reproductive strategies (e.g. Käär et al., 1998; Bird, 1999; Borgerhoff Mulder & Rauch, 2009; Schacht & Borgerhoff Mulder, 2015; Schacht & Smith, 2017; Lawson et al., 2021; Akurugu et al., 2022; Baraka et al., 2022). In recent years, interest has grown in examining local sex ratios and their effect on reproductive decision-making and bargaining power in romantic relationships (e.g. Abramitzky et al., 2011; Francis, 2011; Wei & Zhang, 2011; Lainiala & Miettinen, 2013; Schacht & Borgerhoff Mulder, 2015; Porter, 2016; Uggla & Mace, 2017; Schacht & Smith, 2017), and scholars have extended the theory to test predictions on how sex ratios may influence gender norms (Guttentag & Secord, 1983; Grosjean & Khattar, 2019; Brooks et al., 2022).

In this article, we propose a revision of sexual conflict theory when applied to humans, and discuss important considerations that are essential to understanding the relationship between human sex ratios, bargaining power and gender norms (Table 1). Here we define gender norms as social norms, rules or ideals that govern what counts as socially acceptable and virtuous behaviour and that apportion resources, roles, power and entitlements based on (perceived) sex (Ridgeway & Correll, 2004; Cislaghi & Heise, 2020). The term social norm is used to describe conventions or common behaviours in a particular community, but many norms are also injunctive or prescriptive; they refer to moral values and societal standards (Bicchieri, 2005). Social norms are thought to have evolved because they render the actions of others more predictable and thereby facilitate coordination between community members (O'Connor, 2019). Violation of norms can be costly because it can lead to miscoordination with cooperative partners, or to punishment in reaction to deviance from prescriptive norms. Gender norms pertain to behaviour related to marriage, sexual divisions of labour, respectful conduct and other forms of behaviours that are dependent on one's sex. They are often not merely descriptive, but prescribe ‘morally right’ behaviour. While theoretically gender norms could culminate into complete gender egalitarianism, more typically across cultures they describe a gender hierarchy in which men have more power and higher status than women (Schneider & Gough, 1961; Rosaldo, 1974; Smuts, 1995).

The evolution of social norms is an important topic of study in research on cultural evolution. Cultural evolution theory has been fruitful in showing the importance of biased transmission in the spread and maintenance of cultural traits (Boyd & Richerson, 1985, 2002; Henrich & Boyd, 1998; McElreath et al., 2003; Efferson et al., 2008; Mesoudi, 2011b), but limited consideration has been given to power dynamics and their role in the spread of ideas (Singh et al., 2017; Cofnas, 2018) and this field of inquiry has somewhat ignored gender (Lawson et al., 2023). Instead, social influence is mostly considered in the form of deference and voluntary copying of the behaviour of high-status individuals rather than coercion by those in power. In order to gain a deeper understanding of how gender norms spread, persist and change over time, and of how sex ratios and gender norms interact to affect gendered bargaining dynamics, sexual conflict theory and cultural evolution theory need to be better integrated and applied together.

In this review we summarize sexual conflict theory and discuss how it has been used to understand the influence of sex ratios on the reproductive behaviour of non-human animals. Next, we examine how in human populations, sex-ratio effects on gendered bargaining power can interact with culturally specific gender norms in important ways. We propose that to understand this complex relationship, we need to consider gender norms both as products of sexual conflict and variables affecting sexual conflict by constraining women and men's market values and their freedom of choice in reproductive decision-making. Importantly, gender norms can directly affect sex ratios and at the same time affect how people respond to market dynamics that are a product of those sex ratios. We argue that an integration of models of cultural evolution with sexual conflict theory can help to elucidate these interactions, improving our understanding of sexual conflict in humans.

**Table 1.** Glossary

ASR	Adult sex ratio, ratio of adult men to women in a given population. High sex ratios refer to populations where men outnumber women; low sex ratios refer to populations where women outnumber men (Queller, 1997; Jennions & Fromhage, 2017).
OSR	Operational sex ratio, ratio of reproductively available males to females, which excludes juveniles, pregnant or lactating females and post-reproductive individuals (Emlen & Oring, 1977; Jennions & Fromhage, 2017).
Cultural evolution theory	Theoretical framework in evolutionary anthropology that hypothesizes that cultural traits, like social norms, are subject to processes similar – but not identical – to Darwinian evolution as traits vary and some are more likely to spread than others. At the micro-level, this theoretical framework considers how cultural traits are passed on between individuals, such as through biased social learning.
Gender norms	Social norms, rules or ideals that govern what counts as socially acceptable and virtuous behaviour and that apportion resources, roles, power and entitlements based on (perceived) sex (Ridgeway & Correll, 2004; Cislaghi &Heise, 2020). Gender norms prescribe behaviour related to marriage, divisions of labour, respectful conduct, and other forms of behaviours depending on one's (perceived) sex.
Gender ideology	Ideologies are collections of social norms or ‘cultural beliefs that justify particular social arrangements, including patterns of inequality’ (Macionis, 2010: 257). Gender ideologies then refer to collections of norms that justify a particular hierarchy (or lack of hierarchy) between people on the basis of their (perceived) sex.
Gendered conflict	Sexual conflict as applied to human behaviour, where women and men's conflicting interests cannot be reduced to sex-based biological differences, but are shaped by cultural practices and gender norms (Lawson et al., 2023).
Sexual conflict theory	Theoretical framework in evolutionary biology that seeks to explain how conflicts of interest between females and males result in adaptations over evolutionary time, including behavioural flexibility within the lifespan of individuals.
Adaptive lag	A period of mismatch when adaptations have not yet caught up with current selection pressures.
Mate guarding	Behaviour aimed at maintaining exclusive sexual access to a sexual partner, for example by physically preventing a partner from interacting with other potential partners. In humans, behaviours such as preventing a partner from leaving the house or limiting a partner's financial independence may be interpreted as mate guarding.

## 2. Theory and applications of sexual conflict theory and sex ratios in non-human animals

Research on non-human animals shows that female and male interests may diverge over shared physiological (Trivers, 1972; Kokko & Jennions, 2008), morphological (Bonduriansky & Chenoweth, 2009) or behavioural traits, such as the number of matings (Galliard et al., 2005), control over fertilization (Parker, 2006) and levels of parental investment (Arnqvist & Rowe, 2005). Sexual conflict theory posits that the degree of bargaining power individuals have in conflicts of interest depends on the relative value of all possible conflict outcomes for each partner, which includes the opportunities each has outside the current interaction. Sexual conflicts of interest are expected to be resolved in favour of the party with a better bargaining position.

A crucial factor for determining bargaining power in sexual conflict is the quality and number of alternative partners available to each party. Holding all else equal, the party with more alternative reproductive opportunities has more leverage to achieve their interests. A classic example of sexual conflict is parental investment: when two individuals reproduce, both are interested in the survival of their offspring, but either party may prefer that the other provide the parental investment required so they can spend their own time and energy pursuing other reproductive opportunities (Trivers, 1972; McNamara et al., 2000; Kokko & Jennions, 2008). The individual with better options outside the current partnership has a better ‘fallback position’ and therefore is in a stronger bargaining position. They will, on average, see the conflict resolved closer to their optimal outcome (Clutton-Brock, 1991; Parker, 1979; Arnqvist & Rowe, 2005; Kokko & Jennions, 2008).

At the level of the population, the ratio of males to females is strongly associated with the reproductive options of each sex, and the significance of sex ratio as a determinant of bargaining power in sexual conflict has been exemplified in numerous experimental and observational studies (Carroll, 1991; Székely et al., 2006; Karlsson et al., 2010; Liker et al., 2013; Carmona-Isunza et al., 2015; Eberhart-Phillips et al., 2018).

Sex ratios are usually operationalized as the ratio of males to females in a population, where high sex ratios reflect a bias towards more males and low sex ratios a bias towards more females. There are several sex ratios that are commonly used in these studies. One is the adult sex ratio (ASR), the ratio of reproductively aged males to females. Another is the operational sex ratio (OSR), the ratio of reproductively available males to females, which excludes pregnant, lactating and post-reproductive individuals (Emlen & Oring, 1977) and in humans sometimes excludes married people. The ASR and OSR do not always track each other because of the higher temporal variation of the latter, especially in smaller populations (Carmona-Isunza et al., 2017; Jennions & Fromhage, 2017). In addition, because the time and energy individuals spend on reproduction is itself a product of sexual conflict dynamics (Kokko & Jennions, 2008; Kappeler et al., 2022), which ratio is most relevant depends on the reproductive biology of the species, and on the phenotypic trait and evolutionary time-scale being studied (Jennions & Fromhage, 2017). For example, when testing for sex-ratio effects on male mate guarding behaviour in primates, the ratio of adult males to estrous females may be most relevant, but when examining sexual conflict over parental investment, the OSR is a dependent rather than an independent variable (as which sex spends time in and out of the mating market is the subject of conflict) and the ASR may be more appropriate. The ASR is usually a suitable measure for considering behavioural plasticity and short-term responses to environmental cues in within-species comparisons, and is the measure that is used in most contemporary human studies (Kappeler et al., 2022).

One important domain of sexual conflict is parental investment. Following the principle of allocation, organisms have limited time and energy available to spend on reproduction. In sexually reproducing species a trade-off exists between allocating time and energy towards behaviours aimed at accessing sexual partners and behaviours associated with caring for existing offspring, as the same resource unit cannot be spent on both (Trivers, 1972). This trade-off can result in a conflict of interest between sexual partners over parental investment, where either partner is better off if the other provides the investment needed to produce surviving offspring so that their own resources are free to be spent on mating effort (Clutton-Brock, 1991; Székely & Cuthill, 2000).

The extent to which individuals can improve their fitness through investing time and energy in mating or parenting behaviour depends on species-level, population-level and individual factors. For example, species vary in the amount of parental care required to raise offspring to adulthood, population-level differences in environments may lead to variation in the costs of parental investment and mating effort, and individuals’ reproductive opportunities are partly determined by their ability to compete for mates with others of the same sex. At the population level, the density and ratio of adult males to females shapes the relative payoffs of mating and parenting behaviours, as it determines the number of potential sexual partners and the severity of competition between members of the same sex. For example, when the sex ratio is skewed, mate scarcity can make it more profitable for the abundant sex to devote energy into maintaining reproductive access to a current mate rather than into competing for additional mates (Kokko & Jennions, 2008). To increase their chances of mating, members of the abundant sex can respond to an unfavourable sex ratio by appealing to the preferences of the rare sex, such as by providing more parental care. In addition, the opportunity costs of parental investment go down as the relative benefits of searching for mates decrease owing to strong mating competition. Members of the rare sex can drive a harder bargain in situations of sexual conflict, for example forcing their mate to provide more care while they reenter into the mating pool. We should expect ASR-responsive flexibility in reproductive strategies to occur in species where at least some paternal care is present, and where there is limited reproductive skew (when few males account for most or all offspring in a population, the effect of sex ratios on bargaining power will be minimal). Furthermore, the relationship between reproductive strategies and ASR is dynamic. For example, the mortality risks of parenting and mating strategies can affect the ASR. If increased mating effort leads to higher mortality, the sex ratio becomes more extreme because of a self-reinforcing process where the rarer sex (which would benefit the most from mating effort) becomes even rarer (Kokko & Jennions, 2008).

Shorebirds (*Scolopaci* and *Charadrii spp.*, sandpipers, plovers and allies) have provided an informative model for investigating the relationship between sex ratios and mating and parenting behaviour (Székely et al., 2023). Shorebirds exhibit broad inter-specific variation in mating systems and in the ability of both sexes to provide full parental care on their own, but are generally similar in ecology and variation in sex ratios as result of sex-biases in juvenile survival (Bennett & Owens, 2002; Székely et al., 2006; Liker et al., 2013; Carmona-Isunza et al., 2015; Eberhart-Phillips et al., 2018). In line with predictions, research on these species has found that although sex differences in parental investment cannot be fully explained by considering the ASR alone, female-biased adult sex ratios are linked with more female care and higher levels of polygyny (Liker et al., 2013; Eberhart-Phillips et al., 2018). In species with male-biased adult sex ratios, males are more likely to provide the bulk of parental care, female multiple mating is more common and females have more showy plumage, a trait that is generally associated with investment in mating competition (Liker et al., 2013). A competitive mating market can thus motivate individuals of the abundant sex to provide more parental care to existing offspring (Székely et al., 2023). Similar evidence for parenting behaviour as a flexible response to ASR has been observed in other taxa, such as rails (Maynard Smith & Ridpath, 1972), cichlids (Grüter & Taborsky, 2005) and dung beetles (Rosa et al., 2017).

Evidence in non-human primates for a sex ratio-effect on reproductive strategies is limited, as direct paternal care is absent in most species (Rosenbaum & Silk, 2022), but some supportive evidence comes from callitrichids. Callitrichids are known to have flexible mating systems. While monogamy is most common, polyandrous bonds are more prevalent when the sex ratio is high; under these circumstances one breeding female may be supported by two caring males (Goldizen, 1987; Dunbar, 1995). Breeding pairs may receive help from the male's brother, who may have little chance of siring offspring himself and for whom the opportunity costs of caring are small.

Mating systems can thus be sensitive to a population's ASR, as it affects both the value of parenting and mating behaviours for each sex, as well as their bargaining power (Székely et al., 2014). However, appealing to the preferences of members of the rare sex is not the only possible response to an unfavourable sex ratio. Sexual conflict theory also predicts that males can use coercion to regain bargaining power lost through a skewed sex ratio, as has been observed in many taxa. Higher ASR is associated with increased rates of male-to-female aggression in crab spiders (*Misumena vatia*; Holdsworth & Morse, 2000), common lizards (*Lacerta vivipara*; Galliard et al., 2005), monk seals (*Monachus schauinslandi*; Johanos et al., 2010) and possibly feral horses (*Equus ferus caballus*; Regan et al., 2020), although not all studies have found evidence for a sex-ratio effect (e.g. Head & Brooks, 2006; Baniel et al., 2017). Aggression used to obtain sexual access to females against their will is prevalent in multiple primate species (Smuts, 1992; Muller & Wrangham, 2009; Muller et al., 2011), but there is less evidence that this behaviour is responsive to ASR in primates. Among chimpanzees (*Pan troglodytes*) at Ngogo in Uganda, females received higher rates of aggression from males when the OSR was higher (Watts, 2022), but it is unclear whether this result indicates that males increased their aggressive behaviour towards females when they had many competitors or whether aggression received by females was proportional to the greater number of males present. The latter seems to be the case among mountain gorillas (*Gorilla gorilla beringei*) studied by Robbins (2009). Here the number of males present was not associated with rates of male-to-female aggression, but females did receive more threatening behaviours (chest-beating) when they were in a multi-male vs. single-male group. Similarly, the OSR does not predict how often female chacma baboons (*Papio ursinus*) are subject to aggression by males in their group (Baniel et al., 2017). We are not aware of studies testing for a sex-ratio effect on male-to-female aggression among bonobos (*Pan paniscus*). However, Fruth and Hohmann (2003) found that although female bonobos with sexual swellings are preferred targets of male aggression, male-to-female aggression is not dependent on the number of females in the group exhibiting swellings. Overall, the current literature does not show much support for consistent sex-ratio effects on male-to-female aggression in our closest relatives.

Mate guarding is another tactic used to monopolize the reproduction of a partner when strong competition owing to a male-biased ASR makes finding a new mate unlikely (Harts & Kokko, 2013). Adult sex ratio-sensitive mate guarding has been observed among males of some populations of soapberry bugs (*Jadera haematoloma*; Carroll & Corneli, 1995), water striders (*Gerris buenoi Kirkaldy* and *Gerris lacustris*; Rowe, 1992; Vepsalainen & Savolainen, 1995), beetles (*Lethrus apterus*; Rosa et al., 2017), various crustaceans (Wada et al., 1999; Mathews, 2002; Karlsson et al., 2010; Takeshita & Henmi, 2010) and Soay sheep (*Ovis aries*; Clutton-Brock, 2016: 632). Mate guarding is also common in some primate species, such as baboons (*Papio cynocephalus*; Bulger, 1993), sifakas (*Propithecus verreauxi*; Mass et al., 2009), mandrills (*Mandrillus sphinx*; Setchell et al., 2005) and chimpanzees (Watts, 1998). Again there is little existing research testing whether this behaviour in primates is sensitive to sex ratios, and the samples in existing studies are small (usually consisting of one or two groups where ASR varies temporally). In one longitudinal study of Japanese macaques (*Macaca fuscata*), Takahashi (2001) was able to capture variation in male mating behaviour in relation to temporal changes in OSR. He found evidence that resident males more often violently interfered with floating males’ mating attempts in periods when the sex ratio is more male biased, although this observation could simply mean that mate guarding was less successful when competition was more fierce. Perhaps the effect of sex ratios on mate guarding behaviour is limited in primates because other population- and species-level factors are more important in determining its payoffs. For example, mate guarding often prevents effective foraging and can increase predation risk (Clutton-Brock, 2016), and these costs are expected to vary between environments. Mate guarding also is more costly when ovulation is inconspicuous; the absence of a clear signal of females’ fertility makes it difficult to determine when guarding will pay off (Clutton-Brock, 2016: 480–482). The low reliability of bonobos’ sexual swellings as a signal for ovulation (Reichert et al., 2002; Douglas et al., 2016) may therefore partly explain why mate guarding and male-to-female aggressive behaviours are rare compared with chimps.

Mate guarding can pave the way for paternal care by decreasing the opportunity costs of care as active guarding places males in closer proximity to their offspring. Several scholars have suggested that male mate guarding preceded the evolution of pair bonding in mammals (Lukas & Clutton-Brock, 2013), and more specifically in humans (Schacht & Bell, 2016; Loo et al., 2017), but see Gavrilets (2012) for an alternative view. Loo et al. (2021) hypothesize that mate guarding as a response to high sex ratios may have preceded the high level of paternal care common in callitrichids.

This summary of current research shows that biased sex ratios can drive sexual conflict by affecting the reproductive opportunities of individuals in the population and are an important source of bargaining power in situations of sexual conflict. Having fewer reproductive options changes the value of different reproductive strategies for both sexes, and can lead to individuals of the more abundant sex adhering more to the preferences of potential mates. Alternatively, males have been shown to respond to an unfavourable bargaining position with physical coercion of females. Evidence for this sex ratio-effect is weak in primates. This could be due to the difficulties in obtaining large sample sizes of populations that vary in ASR but where other factors affecting mating behaviour are held constant (or are statistically accounted for). Alternatively, sex-ratio effects may be completely absent when other ecological factors play a larger role in determining the costs and benefits of parenting and mating strategies.

## 3. Applying sexual conflict theory and sex ratio predictions to human populations

Following theory and findings in non-human animal populations, sex ratios are also predicted to influence the payoffs of different reproductive strategies in humans. Going one step further, sex ratios have also been hypothesized to affect the status and treatment of women, a point first made by Guttentag and Secord (1983). They argued that while men hold ‘structural’ power in patriarchal societies, women can gain ‘dyadic’ power from a male-biased marriage market, thereby gaining higher status within the domain of the household and family. Guttentag and Secord's (1983) predictions on sex ratio-effects somewhat overlap with evolutionary hypotheses, but where Guttentag and Secord do not question the origins of men's ‘structural power’, evolutionary anthropologists have argued that patriarchal norm systems are the result of a long evolutionary history of sexual conflict (Smuts, 1995). According to this view, the reproductive biologies of females and males result in men's greater interest in control over women's reproduction. Over evolutionary time, this resulted in gender norms that have justified men's dominance over women. Variation in women's status and bargaining power within marriage however is, similar to Guttentag and Secord's concept of dyadic power, hypothesized to depend on their alternative reproductive options, which are partly determined by the population's sex ratio.

Many studies testing sex ratio hypotheses on human reproductive behaviour have suffered from methodological issues and yielded mixed results (reviewed by Schacht & Smith, 2017). One common issue with studies on sex-ratio effects highlighted by Pollet et al. (2017) is the use of aggregate national-level data (such as in South & Messner, 1987; Diekmann, 1992; Barber, 2000; Kruger et al., 2010). Adult sex ratio is non-normally distributed at the national level, which can result in a strong but spurious effect of outliers. These analyses also often disregard the confounding effects of geographic clustering and shared cultural histories, which can lead to similarities between nearby populations that may mistakenly be interpreted as the result of shared ecological factors (Pollet et al., 2017). More fine-grained studies have been able to avoid some of these issues by relying on localized sex ratio data, and these studies have been more successful in convincingly identifying cases in which biased sex ratios affect reproductive strategies in line with sexual conflict predictions. We will now review empirical work that uses local ASR data to study these topics, and then discuss how this work is limited by not seriously considering the role of cultural norms.

Many studies find that romantic relationships are more stable in areas when the ASR is male-biased, supporting the sexual conflict prediction that men are more motivated to stay with current partners (and perhaps invest more in their children) when alternative partners are scarce (e.g. Pedersen, 1991; Pouget, 2017; Uggla & Mace, 2017; Uggla & Andersson, 2018; Grosjean & Khattar, 2019). Anthropologists have also looked for evidence of increased bargaining power of members of the rarer sex. For example, Pollet and Nettle (2008) examine men's probability of marriage in a representative US sample from 1910. They found a positive interaction effect between socioeconomic status (SES) and local ASR on marriage; higher SES was a more important predictor for the probability of marrying in states where the ASR was more male-biased. This could indicate women's better ability to make demands on partners owing to their better bargaining position, although this is not universally true. In a study of cohabitation patterns in Northern Ireland, Uggla and Mace (2017) find that women are more likely to cohabitate in areas with higher ASR, but do not find evidence that men's SES becomes more important as a predictor of their probability to be in a stable relationship when the ASR rises.

Schacht and Smith (2017) test for sex-ratio effects in a historical sample from nineteenth-century Utah, where the population-level sex ratio was male-biased owing to the immigration of Mormon men, while local differences correlated with variation in male mortality rates and child sex ratios, as well as the incidence of polygyny. Comparing 206 districts with varying sex ratios, their results show that men married later in districts with more male-biased ASR. Schacht and Smith interpret this as evidence for women's higher bargaining power when they are scarce, as women are expected to prefer older partners who are in a better economic position. In a study on the relationship preferences of women and men in French Guyana, Schacht and Borgerhoff Mulder (2015) find that men hold stronger preferences for long-term relationships in villages with higher ASR. Building on sexual strategies theory (Buss & Schmitt, 1993), they assume that, on average, women have a stronger interest in long-term vs. short-term sexual relationships compared with men, and therefore argue that this result may indicate that men adjust their preferences to meet women's interests when they have a poorer market position. An alternative interpretation of their result is that men's varying preferences for stable relationships may reflect ASR-sensitive mate guarding. Another study shows that in Chinese regions where the ASR is higher, families with sons save more money (Wei & Zhang, 2011). These savings may function as parents’ investment in the marital position of their sons where women (and their families) can make high demands on potential grooms. Also in China, Porter (2016) reports that men smoke and drink less under more male-biased sex ratios, and proposes that this is an effort to make themselves more attractive as romantic partners. Similarly suggesting men's increased efforts to appease potential partners, Francis (2011) finds that when the ASR in Taiwan increased sharply after 1950 as male soldiers and refugees arrived from China, children were more highly educated in regions where sex ratios were higher. These findings are explained as evidence of men's higher parental investment in response to women's increased bargaining power. Finally, historical sex ratios in Australia have been found to predict current-day attitudes towards women's work. In areas that historically had high sex ratios owing to the in-migration of European male convicts, women and men today are more likely to be in favour of traditional gender roles in which women stay at home while men are the sole breadwinners (Grosjean & Khattar, 2019). Women in areas with historically high ASR also report more leisure time, and men provide a larger share of the household income. The authors interpret these results as evidence of women's greater bargaining power, arguing that these historical bargaining dynamics are still reflected in today's norms.

One methodological issue in many of these studies is the comparison of only a few subpopulations: often the behaviours or attitudes of people from only a few towns, regions or cities are compared, and the possibility that factors beyond ASR confound study results is rarely considered. Cultural differences may covary with the ASR in consequential ways. For example, Uggla and Mace (2017) report that in their Northern Irish sample ASR is on average higher in rural areas as women more often leave the countryside to move to cities. This suggests that the co-occurrence of high ASR and higher cohabitation rates could at least in part result from cultural differences between urban and rural areas, and gender norms could confound the relationship between ASR and cohabitation rates if women migrate because of a preference for the qualities of urban men. In the Taiwanese study (Francis, 2011), the observed correlation between ASR and children's education could be caused by other factors. For example, education may have been more readily available in those areas that attracted more migrants.

Another important issue is the interpretation of various measures of bargaining outcomes. For example, social scientists often regard women's lower fertility as evidence of their higher bargaining power, drawing from the assumption that women tend to prefer fewer children, and when they have greater autonomy are better able to exert preferences over ideal family size (Bankole & Singh, 1998; Borgerhoff Mulder, 2009; Snopkowski & Sear, 2013). Adaptive logic used to explain this difference is that women face greater reproductive costs than men do, and therefore men can afford to have higher fertility preferences (Penn & Smith, 2007; Borgerhoff Mulder & Rauch, 2009). However, Moya et al. (2016) point out that this assumption may be faulty, as men and women do not necessarily have conflicts over ideal family size, and men also suffer costs to reproduction. One of the key costs that Moya et al. highlight relates to sex ratios and bargaining power. The assumption that men can easily replace a wife who dies in childbirth does not take into consideration that men compete for access to wives, and this competition will be particularly strong when the ASR is male-biased. Men may be better off adjusting their fertility preferences to match that of their wives (e.g. with longer interbirth intervals or lower overall fertility) in order to reduce her reproductive costs.

In another example, men's higher paternal investment has also been attributed to women's increased bargaining power (Francis, 2011). While this is one possibility, the opportunity costs of paternal care decrease when partners are scarce, so this behaviour is therefore not conclusive evidence of women's higher bargaining power. A similar point complicates the finding of higher endorsements of traditional gender roles in Australian regions that experienced high sex ratios in the past (Grosjean & Khattar, 2019). Grosjean and Khattar understand men's higher contributions to the household income and support for traditional gender roles as a result of women's high bargaining power. We suggest another, and perhaps more likely, explanation: that men's willingness to be the sole breadwinner reflects a mate guarding strategy. Men may have preferred for their wives to stay at home when they were faced with strong competition from other men in an unfavourable marriage market.

These examples illustrate how the interpretation of bargaining outcomes as favouring men or women is not always clear-cut. This is important, because theory and findings from the animal literature suggest that partner scarcity can lead to either coercive or conciliatory behaviour. Currently the most conclusive evidence for people's use of coercion in response to unfavourable sex ratios comes from studies on intimate-partner violence (IPV) against women. Intimate-partner violence has been theorized as a male strategy to seek control over a female romantic partner in situations where she has a higher market value than he has (Macmillan & Gartner, 1999; Kilgallen et al., 2021). Research in India (Bose et al., 2013) and several US subpopulations (Avakame, 1999; D'Alessio & Stolzenberg, 2010; Vanterpool et al., 2021) indicates that IPV is more prevalent in regions with male-biased sex ratios. Here the work of Bose et al. (2013) is most convincing. They use survey data from married women in the 2005–2006 Indian National Family Health Survey to test the effect of ASR (measured at the level of the village or neighbourhood block) on their reports of husband-to-wife violence. India's male-biased ASR is the result of a strong son bias that has led to biased sex ratios at birth and gender differences in child mortality in many parts of the country. Bose et al. (2013) find a small positive association between ASR and physical, sexual and psychological IPV. Other studies have found similar results using data from the US. For example, D'Alessio and Stolzenberg (2010) use city-level data on male-to-female intimate-partner violent crime rates in the US (which include murder, abduction, rape, sexual assault, etc.) as their dependent variable. They find that more IPV crimes occur in cities with male-biased sex ratios, although the very high coefficients in their model results are difficult to interpret and suggest their models may be overfitted or lack appropriate controls for variables correlated with ASR (D'Alessio & Stolzenberg, 2010: table 2). Furthermore, it is unclear whether their results could be explained by a higher prevalence of marriage and lower divorce rates generally, factors that have been associated with high sex ratios (e.g. Angrist, 2002; Abramitzky et al., 2011; Brainerd, 2017), rather than an increase in the rate of IPV in romantic relationships. In a similar study, Vanterpool et al. (2021) recruited Black women from the US on MTurk, as Black communities in the US experience wide variation in ASR resulting from high incarceration rates in some areas. They too find an association between perceptions of high sex ratios and experiences of IPV, but their study is limited by the use of perceptions of both variables rather than demographic data.

As these studies show, it is difficult to ascertain what causes the observed relationship between the ASR and IPV, especially in settings where biases in ASR result from patriarchal norms. In India for example, regional differences in ASR exist owing to the occurrence of cross-cousin marriage, matrilocality and matrilineal inheritance in some parts of the country. These cultural traits are associated with a lack of son preference as well as with higher status and better treatment of women (Dyson & Moore, 1983; Chakraborty & Kim, 2010), leaving the possibility that differences in attitudes towards women's status explain variation in both ASR and the prevalence of IPV, without a causal link between the two.

**Figure 1.**
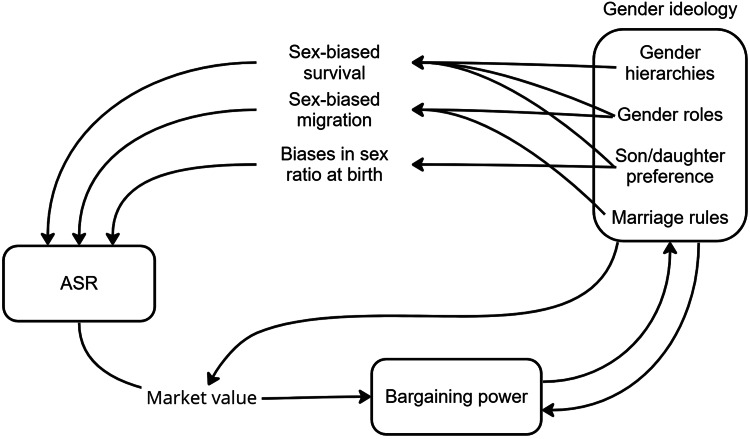
Schematic representation of the relationship between ASR and gender ideology.

While locally contextualized studies have shown the potential for ASR to interact with reproductive strategies, we find that they rarely address the complex gender dynamics that form a pathway between ASR and resulting reproductive strategies. Examining the relationship between ASR, gender norms and reproductive strategies may help us to understand why simple mating market predictions sometimes fail to explain variation in reproductive strategies and bargaining power, as is also suggested by Schacht and Smith (2017). In the remaining part of this review, we revise existing theory to incorporate the role of gender norms and cultural evolution in human sexual conflict. We focus on five important points that address how gender norms interact with sex ratios to affect mating market dynamics, reproductive strategies and gendered bargaining positions: (1) gender norms do not always reflect bargaining outcomes but are subject to their own selective processes; (2) gender norms create bounds on who can reproduce with whom and what is valued on the marriage market; (3) gender norms have the potential to constrain people's reproductive decision-making power in a way that is unequal between genders; (4) biases in local ASRs themselves are often the result of gender norms; and (5) gender norms affect how individuals respond to biased sex ratios. Collectively these points show that sex ratio dynamics – and sexual conflict more broadly – in humans are deeply interlinked with gender norms (see Figure 1), and that the interpretation of sex ratio biases as favouring men or women is not always clear-cut. This fits with theory and findings from the animal literature, which suggest that partner scarcity can lead to either coercive or conciliatory behaviour. Here we have shown some examples that are clearly coercive (e.g. IPV) and examples that are more conciliatory in nature (e.g. paternal investment), but most of the studies we have highlighted have data that can be interpreted multiple ways, and reflect the need for deeper contextualization of history, culture and demographics.

We conclude this paper by arguing that human sexual conflict may be better termed ‘gendered conflict’ (Lawson et al., 2023), because it can only be understood by explicitly modelling the role of gender norms and by integrating models of cultural evolution.

### 3.1. Gender norms are a product of sexual conflict as well as other evolutionary processes

Evolutionary anthropologists have argued that gender norms are the result of a long evolutionary history of sexual conflict (Smuts, 1995; Hrdy, 1997). According to this view, the reproductive biologies of females and males result in males’ higher interest in control over females’ reproduction than vice versa. Smuts (1995) argues that patriarchal norms that justify men's dominance over women have evolved as a continuation and extension of male efforts to control female reproduction, common in the animal kingdom and specifically in mammals and primates. Importantly, ‘human males are not “genetically programmed” to coerce and control women, and […] women are not “genetically programmed” to accept subordinate status’ (Smuts, 1995: 21). The evolution of patriarchy refers to the *cultural* evolution of social norms prescribing male dominance, and Smuts argues that human residence patterns, male control over resources and the uniquely human ability to convey information through language have enabled greater control of men over women than is possible in other animals (Smuts, 1995). Gender norms are socially enforced by group members (and often by the state) through punishment or social exclusion (Egan & Perry, 2001; Blakemore, 2003; Parrott, 2009; Skočajić et al., 2020). Like other social norms, gender norms may adjust to changing community members’ interests in norm-governed behaviours, as well as to their bargaining positions (Singh et al., 2017). Sex ratio dynamics can therefore influence gender norms by changing individuals’ interests. For example, extreme sex ratios can motivate a tolerance of polygamy (Starkweather & Hames, 2012), and in other cases have led to the relaxation of exogamy rules (Mishra, 2013; Larsen & Kaur, 2013). However, there are various reasons why norms do not always reflect a bargaining outcome of the optimal strategies of individuals in a social group.

First, social norms are often subject to a period of mismatch when adaptations have not yet caught up with current selection pressures. Such ‘adaptive lags’ may be caused by a number of processes. One example is group members’ efforts to coordinate behaviour by adhering to social norms. Social norms structure human behaviour in such a way that makes it predictable and facilitates smooth coordination between group members. People who deviate from the norm could experience costs simply from miscoordination (Young, 1993; Centola & Baronchelli, 2015; O'Connor, 2019). In China, women who pursue higher education risk not finding a partner, as they often seek men who are willing to leave behind traditional roles of the husband as breadwinner and the wife as homemaker, while many men prefer women who are less educated than themselves and will take on the role of homemaker (Ji, 2015; Hong Fincher, 2016). Such mismatches in partner preferences may occur when one tries to depart from common gender norms. In addition, once a norm becomes an important part of the moral code of a group, deviance from the norm can lead to important social costs, such as exclusion or punishment by other group members. Deviance from culturally dominant gender norms also can result in negative health outcomes via negligence or violence (Macmillan & Gartner, 1999; Weber et al., 2019; Kilgallen et al., 2021). The coordination benefits of norm adherence as well as the social costs of deviance therefore can lead to cultural inertia that results in a disconnect between a group's norms and group members’ optimal behaviour. Because of this, cultural phylogeny constrains changes in social norms, as is probably the case for marriage norms: in a phylogenetic analysis of cultural groups included in the Standard Cross-Cultural Sample, the cultural history of populations explained twice the variance in local rules on polygyny and in the occurrence of polygyny as relevant ecological predictors (Minocher et al., 2019). Here adaptive lag is one possible explanation for the lack of divergence between related groups.

Second, norms themselves are subject to evolutionary processes that are separate from the payoffs they provide to group members. Cultural evolution theory hypothesizes that cultural traits, among which social norms, are subject to processes similar but not identical to Darwinian evolution, as traits vary and some are more likely to spread than others. At the micro-level, this theoretical framework considers how cultural traits are passed between individuals, such as through biased social learning (Boyd & Richerson, 1985; Boyd et al., 2011; Mesoudi, 2011a; Richerson & Boyd, 2005). For example, the rate at which norms spread depends on the cultural ‘models’ displaying those norms. Various studies have shown that people are more likely to adopt cultural traits held by high-status individuals (Henrich & Gil-White, 2001; Atkisson et al., 2012; Chudek et al., 2012). Common norms may also spread more easily by virtue of their popularity when people conform to the majority (Henrich & Boyd, 1998; Efferson et al., 2008; Muthukrishna et al., 2016; Hagen & Scelza, 2020). As an example, practices of female genital cutting (FGC) are thought to have culturally evolved from conflicting interests between women and men by limiting women's desire for extra-pair sex while increasing their husbands’ paternity certainty. However, recent research on this topic shows that while FGC is clearly harmful to women, its effect on women's sexual behaviour and thereby men's paternity certainty may be limited (Howard & Gibson, 2019). Rather than being an outcome of continued sexual conflict, frequency-dependent copying may drive the persistence of FGC (Howard & Gibson, 2017). Specific to marriage norms, Henrich et al. (2012) have suggested that group-level benefits to monogamous marriage drove the spread of monogamy to many populations around the world. In their argument, social enforcement of monogamous marriage is argued to reduce intrasexual competition, result in more men being married and higher paternal investment. Henrich et al. (2012) argue that norms prescribing monogamy may have increased the economic prosperity of monogamous people, and that this prosperity helped spur its spread to other populations. If correct, this implies that marriage rules, like other gender norms, can spread as a result of biased cultural transmission.

In summary, gender norms are thought to result, in part, from an evolutionary history of sexual conflict. While the norms in a population are probably subject to change depending on the interests and bargaining positions of its members, they are also subject to other processes and are therefore unlikely to perfectly reflect sexual conflict-derived bargaining outcomes.

### 3.2. Gender norms constrain someone's value on the market

**Figure 2.**
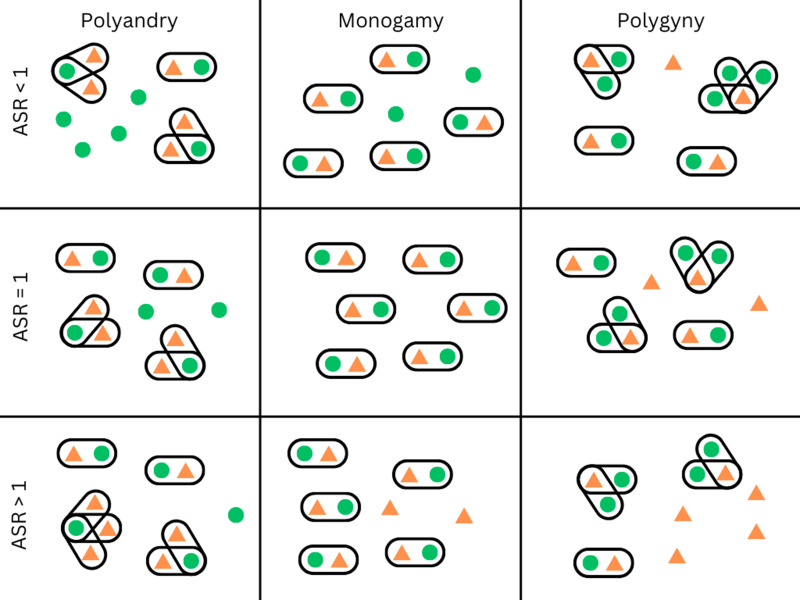
This diagram illustrates the effect of polygyny on partnership opportunities in social groups depending on the local ASR. Green circles indicate women, orange triangles refer to men, and lines around individuals indicate marriages or pair bonds. Rows represent different ASR, and columns vary in their marriage rules. The ASR and local marriage rules interact to determine marriage opportunities. For example, in the upper row the population ASR is female biased. When monogamy is the norm, some women remain without a partner. When men can have multiple partners, this dampens the effect of a female-biased sex ratio (and could even result in some men remaining partnerless). If polyandry occurs, this exacerbates the scarcity of partners for women.

Gender norms both constrain who is on the market and regulate what their value is. Concerning the first, marriage systems are a well-known constraint on reproductive strategies, and interact with ASR in important ways. Whether socially or legally enforced, norms about who is ‘on the market’ and about how they are valued are often not gender-neutral. For example, gender differences may exist in the social acceptance of remarriage after divorce or after the death of a spouse (Whyte, 1978), and the legal minimum age for marriage is lower for women than for men in 43 out of 201 countries (UN Statistics Division, 2013). Furthermore, the acceptance or rejection of polygamy plays a large role in determining the ‘market value’ of women and men. In populations where polygyny is culturally normative, some men have the ability to monopolize the reproduction of multiple women. In populations where the ASR is female-biased, polygyny can increase the demand for women, which – all else equal – could improve their bargaining position (Becker, 1981). The costs to men in this situation may also be minimized, as a female-biased sex ratio can lead to most men being able to have at least one wife (see Figure 2). This is very different from cultures where polygyny is allowed and the sex ratio is equal or male-biased. In these cases, reproductive skew would be exacerbated by the concurrent demographic and cultural restrictions on access to women. In 77% of cultures in the Standard Cross-cultural Sample, men are allowed to have multiple spouses, whereas only 6.5% allow women to have multiple spouses (Whyte, 1978). Today polygyny is legal in 50 countries, and in these countries between 2 and 36% of people live in polygynous households (Pew Research Center, 2019). At least one study examines the level of polygyny in relation to local sex ratios: Pollet and Nettle (2009) find that in Uganda, the percentage of men in polygynous unions tracks the regional adult sex ratio, with more men in polygynous unions when the sex ratio is female-biased. Polyandry can also be affected by sex ratios. Starkweather and Hames (2012) review the literature on non-classical polyandry and find that it is sometimes practised when populations are faced with a particularly male-biased sex ratio. However, strong cultural norms prohibiting polygamy prevent this from happening in many cultural groups. For example, the scarcity of French men after World War II led to higher bargaining power for men, but not to a tolerance for polygynous marriage (Abramitzky et al., 2011). Therefore, while it is possible for polygamy to dampen sex-ratio effects on marriage market dynamics, strong cultural norms and marriage rules can limit this potential.

When the sex ratio is skewed but all else is equal, does polygamy result in higher bargaining power for the abundant sex by increasing their demand, as follows from a marriage market approach (and is suggested by, among others, Becker, 1981)? Importantly, all else is usually not equal. As we argue in this paper, marriage rules, gender norms and sex ratios are all interlinked. Polygyny is often accompanied by a strong gender hierarchy, male-controlled access to resources and reduced decision-making power for women. This issue is further discussed by Grossbard (2015).

Norms regarding beauty standards and gender roles have a strong influence on partner preferences and thereby affect how people are value on the marriage market. Norms regarding gender roles and divisions of labour can also affect bargaining power in a special way: they can create a stronger interdependence among romantic partners who have a family to care for. This is true for gendered divisions of labour common in subsistence-based communities, such as in hunter–gatherer populations where men and women have complementary foraging tasks, as well as the breadwinner–homemaker division of labour that is common in urbanized populations around the world, where men more often work outside the home while women perform more of the childcare labour and other household duties. The types of labour and the roles prescribed to women and men have far-reaching consequences for women's and men's relative dependence on another. Earning an income by working outside the home is a source of power for a variety of reasons, an important reason being decreased dependency on a spouse by improving someone's options outside of a relationship, as is discussed extensively in a large literature in anthropology, sociology, feminist economics and development studies (for example see Schlegel & Barry, 1986; Lundberg & Pollak, 1996; Agarwal, 1997; Kantor, 2003; Vyas & Watts, 2009). On the other hand, men are also reported to respond to women's employment with violent backlash when women's work conflicts with their perceived notions of traditional gender roles (Atkinson et al., 2005; Weitzman, 2014; Cools & Kotsadam, 2017; Kilgallen et al., 2021).

Marriage rules, beauty standards and gender roles are thus three examples of ways in which gender norms have a strong effect on people's value on the marriage market.

### 3.3. Gender norms affect freedom of choice in a way that can override marriage market opportunities

In many populations, women and men do not have the same degree of freedom in reproductive decision-making. Sometimes individuals of only one gender can approach the other with a marriage proposal, allowing the other party only a choice between refusal or acceptance. Parents often influence the choice of spouse for children of both women and men, but cross-culturally men have a say in their own marriage choices more often than women (Whyte, 1978). Parents’ and children's interests often do not completely overlap, especially when high marriage payments are involved or marriage is in another way consequential to a woman's family (Borgerhoff Mulder, 1998; Apostolou, 2009). Bridewealth is subject to market demands for women and can rise under male-biased sex ratios (Francis, 2011), increasing parental interests in controlling a daughter's marital decisions and causing further divergence of parents’ and daughters’ interests. This can have the effect of limiting women's autonomy where their value on the marriage market is high. Here the function of the bridewealth is important. For example, in China bride wealth is skyrocketing owing to male-biased sex ratios (Wei & Zhang, 2011), but much of the bride wealth devolves to the couple (for example in the form of real estate, cars or other valued goods), making it more similar to dowry (Yan, 2003; Wei & Zhang, 2011). Dowries can also be sensitive to the demand for men (e.g. Rao, 1993), but high dowry does not lead to the same level of parental control because dowries often function as pre-mortem inheritance that will be owned by the married couple rather than as a ‘payment’ to the groom's family (Goody, 1976; Gaulin and Boster, 1990).

Second, normative restrictions on pre- or extra-marital sex constrain people's ability to benefit from a favourable marriage market. Premarital sex allows people entering the marriage market to learn about potential partners available to them while extramarital sex can make it easier to switch partners (Buss et al., 2017; Scelza & Prall, 2023). A double standard where men have more freedom to engage in both premarital and extramarital sex is common throughout the world, limiting women's access to information about available partners (Broude, 1980). Once again, however, gender norms can interact with ASR to affect these practices. Himba have a strongly female-biased ASR, but this co-occurs with a long history of matriliny and a pastoralist production system that requires long periods of spousal separation (Scelza et al., 2021). In this setting, while the female-biased ASR may be leading to a marriage market that is more favourable for men, the social history and ecological circumstances have contributed to gender norms that allow women significant sexual autonomy, so that both premarital and extramarital sex are common for women.

Lastly, gender-based differences in the right and ability to initiate divorce (and remarry) potentially have a strong effect on women and men's bargaining power in marriage (Scelza, 2013). Bargaining power derived from a favourable mating market can be nullified if the costs of divorce are high, for example because of legal restrictions or social stigma on divorce (Bargain et al., 2020), and normative restrictions on women's ability to initiate divorce are more common than on men's (Broude & Greene, 1983). In most countries women and men legally have equal access to divorce, but restricted unilateral or fault-based divorce laws in some pose limits on divorce that may disproportionately affect women's ability to leave their husbands. Furthermore, women's prospects after divorce may be further limited by their financial dependence on husbands (Leopold, 2018). Gender-based restrictions on divorce could lead to counterintuitive situations in which a biased sex ratio results in a good bargaining position of one gender when looking for a marriage partner, but very little bargaining power in the marriage itself. These double standards on women's and men's freedom of choice in marriage are expected to lead to an asymmetric relationship between their market value as derived from the ASR and women's and men's bargaining power, where women are often more constrained in their ability to gain leverage from a favourable sex ratio owing to stronger limits on their freedom of choosing partners, initiating divorce and having pre- and extramarital sex.

### 3.4. Gender norms directly affect sex ratios

To test mating market predictions, many studies understandably focus on populations in which the factors resulting in biased sex ratios are exogenous to bargaining dynamics. Many researchers purposefully select populations in which sex-biased migration (Angrist, 2002; Schacht & Borgerhoff Mulder, 2015; Uggla & Mace, 2017; Grosjean & Khattar, 2019), excess male mortality (Jones & Ferguson, 2006; Abramitzky et al., 2011; Brainerd, 2017) or high male incarceration rates (Fossett & Kiecolt, 1993; Cready et al., 1997; Vanterpool et al., 2021) are the main cause of bias in the ASR, rather than the respective status of women and men. However, gender norms themselves in many cases directly influence local adult sex ratios. This can happen through various pathways. Norms enforcing a gendered division of labour can affect the adult sex ratio when some tasks are associated with increased mortality. Hunting and warfare are examples of work typically assigned to men that carries a high mortality risk and that can contribute to biased adult sex ratios (Abramitzky et al., 2011; Brainerd, 2017). Labour migration until recently was more often male-biased, but women constitute a growing part of the world's main migration flows, either following their partners or seeking better employment or education by themselves (summarized by Clarke, 2003).

There are various pathways through which gender norms, and the differential valuation of boys and girls, can affect sex ratios. A preference for either girls or boys can be a strong motivator for parity progression and thereby have important demographic consequences. For example, among Mosuo people in southwest China, where descent practices vary between communities, matrilineal descent practices are associated with an increased likelihood of continued fertility after a son, and patriliny is linked with continued fertility after a daughter (Mattison et al., 2016). In India the last-born child is more likely to be a boy than a girl, and Chaudhuri (2012) estimates that 7% of the births are the result of son preference. In addition, a preference for one gender can lead to inferior treatment of the other, resulting in biased mortality rates. Under-five mortality is biased against girls in the Caucasus and Central Asia, and biased against boys in Uganda, Guinea–Bissau, Uzbekistan and Mongolia (Alkema et al., 2014). At the extreme, sex preferences can result in sex-biased infanticide, with evidence suggesting this practice was once widespread in South and East Asia, Europe and small-scale societies across the globe (Sen, 1990; Divale & Harris, 1976; Smith & Smith, 1994; Mungello, 2008; King, 2014; Dong & Kurosu, 2016). On top of these sources of bias in child sex ratios, access to sex-selective abortion in combination with child-limiting and family-planning policies have led to skyrocketing sex ratios at birth in India and China (Banister, 2004; Zhu et al., 2009; World Bank, 2022).

Although the effects can be somewhat mitigated through sex-biased migration, biased sex ratios at birth generally result in biased ASR in the next generation. Countries where the sex ratio at birth is statistically higher than would be expected in the absence of son-preference account for 38% of today's world population and for 91% of all people living in countries with an ASR over 1.05 (calculation based on population estimates from World Bank, 2022; Chao et al., 2019). These numbers underline the significance of gender norms and their role in the origin of biased sex ratios. This relationship cannot be ignored, especially because of our final point: gender ideology can affect how people may respond to their market value and how market value is translated into gendered bargaining positions.

### 3.5. Gender norms affect how people respond to gains or decreases in their market value

Theory and findings from the non-human animal literature suggest that men have two options in their treatment of women when faced with high competition on the marriage market. They can increase their chances by moving towards the preferences of a (potential) partner, which would indicate a true improvement of women's bargaining position and result in women's higher status. Alternatively, when men's market value is low because of a biased ASR, they can force their bargaining position through coercive tactics such as mate guarding or (threats of) social or physical coercion. Which of these strategies becomes prominent in a particular place is likely to reflect existing gender ideologies. For example, in a population that already strongly enforces men's dominance over women, the latter may be more prominent, while in more gender egalitarian settings conciliatory tactics might be more common. Guttentag and Secord (1983) were among the first to predict a role for gender ideologies in shaping people's responses to biased sex ratios. They argue that under a biased sex ratio, members of the rare sex gain ‘dyadic’ power in romantic relationships. However, they also state that the effect of high sex ratios on women's status will not have the same effect as low sex ratios on men's status, as men disproportionately hold ‘structural power’ in human societies. The combination of these two factors means that the ways that sex ratio imbalances play out are not mirror images of one another. According to Guttentag and Secord, a male-biased sex ratio will lead to an increased valuation of women's reproductive value, but not women's empowerment. Under a male-biased sex ratio, men ‘must treat [their partner] well or run the risk of losing her to another man. But their structural power is sufficient to allow them to place constraints on women's freedoms and impose a sexual morality on them’ (Guttentag & Secord, 1983: 28). This can be seen in many cross-cultural instances of restrictions on female autonomy and sexuality. But more conciliatory responses to a male-biased sex ratio might be more common in places where gender norms are more egalitarian. For example, in a historical US study, Pollet and Nettle (2008) found that in states with a more male-biased ASR, women were able to leverage greater demands, with higher SES men being more likely to be married. Here, men were winning the mating competition by investing.

When it comes to female-biased sex ratios, Guttentag and Secord predict a devaluation of women in society and the relegation of women as mere sex objects. Several studies suggest that men may a higher preference for short-term relationships and uncommitted sex in places where there is a more female-biased ASR, including among US college students (Adkins et al., 2015), in US cities (Kruger & Schlemmer, 2009) and in rural southwestern Guyana (Schacht & Borgerhoff Mulder, 2015). However, gender norms can temper this effect. For example, Himba pastoralists have a strongly female-biased ASR, but also a history of matriliny and strong norms for female sexual autonomy and freedom of movement. Their labour in both production and reproduction is also highly valued. This means that although women have lower market value because they are more plentiful, gender norms that allow for easy divorce and concurrent partnerships mean that women are still able to exert partner choice and leverage the market value they do have in ways that benefit them (Scelza et al., 2019, 2021). The ways in which women respond to unfavourable market conditions may also relate to other factors like post-marital residence patterns and the availability of allocare. For example, where women can rely on others to help care for their children, the costs of reduced male support will not be as great as they are in patrilineal, patrilocal societies where women are more separated from their kin and have less access to alternative forms of support if their partner leaves them.

Where Guttentag and Secord do not question the origins of men's ‘structural power’, evolutionary anthropologists understand patriarchy to be the result of a long history of sexual conflict (Smuts, 1995; Hrdy, 1997). Our perspective is similar in that it predicts that the common gender ideology in a population influences how people respond to their market value as derived from the sex ratio. As reviewed above, responsivity to biased sex ratios has been documented in human populations for both conciliatory and coercive behaviours. We further show that gender norms can affect mating market dynamics. However, more work in this area is needed. In particular, more empirical studies looking at how market dynamics may be responsive to gender ideology would help to test and refine our predictions.

## 4. Discussion

Currently much of the research in the human literature is limited by its reliance on samples from only a few areas with a limited ecological range, making it difficult to separate sex-ratio effects on gender norms from other differences between subpopulations. For example, environmental factors can correlate with sex ratios in a non-causal way and lead to false estimates of sex-ratio effects. Importantly, not all variables interpreted as bargaining outcomes are conclusively indicative of women's or men's preferred bargaining outcome, further complicating this research. In order to effectively study this relationship between sex ratios and bargaining over reproductive strategies, anthropologists must reckon with the endogenous role of gender norms in sexual conflict dynamics. Gender ideology can be an important cause of biases in sex ratios, and probably affect how individuals respond to biased sex ratios. Gender norms thereby have the potential to influence how sex ratios translate to gendered bargaining dynamics, and future work should explicitly take this into account in their predictions as well as in the interpretation of their findings.

In this paper we have argued that the interaction between gender norms and sexual conflict adds a layer of complexity that cannot be ignored when studying sexual conflict in humans. To explicitly foreground this important role of gender norms, we agree with Lawson et al.'s (2023) proposal of the term ‘gendered conflict’ when applying sexual conflict theory to human behaviour. We have provided evidence and suggest hypotheses for some of the myriad ways in which gender norms and sexual conflict interact specifically in the context of sex ratios. As Lawson et al. (2023) discuss, referring to gendered conflict does not negate the central role of sex and reproduction in conflict over evolutionary time, but is useful in that it helps underline the role of social and cultural influences. The term gendered conflict works to disessentialize differences between women and men by underlining the fact that women and men's conflicting interests cannot be reduced to sex-based biological differences, but are very much shaped by gender norms. We further argue that models of cultural evolution are crucial in understanding gendered conflict, while gender norms as well as bargaining and power dynamics are somewhat neglected in cultural evolution theory itself (Singh et al., 2017; Lawson et al., 2023). Sexual conflict theory has been used quite extensively to model and measure bargaining over gender roles and over women's autonomy and decision-making power (e.g. Smuts, 1995; Käär et al., 1998; Borgerhoff Mulder & Rauch, 2009; O'Connor, 2019; Kilgallen et al., 2021). This work centres on the question of how fitness payoffs can affect the spread and maintenance of gender norms and is mostly separate from the literature on cultural evolution theory, which has long studied the evolution of social norms but rarely looks explicitly at gender. Some notable exceptions include a theoretical study of the evolution of sex-biased transmission (Zefferman, 2016) and a study showing FGC as a frequency-dependent behaviour that is associated with increased fitness (Howard & Gibson, 2017). Future studies could go further to use models of cultural evolution will be required to understand how gender norms spread, persist and change over time. These models will need to consider how power dynamics and gendered fitness interests affect these dynamics. At minimum, future studies on sex-ratio effects need to consider the processes leading to biased sex ratios and their relationship to gender ideologies. Where previous research has somewhat neglected the causal role of gender norms or simply avoided populations in which gender norms are an obvious source of variation in ASR, there is opportunity for future research to study these topics in populations where there is a direct link between gender ideology and ASR. Disentangling the complex links between sex ratios, gender norms and gendered bargaining power will be no easy feat. Finding suitable cases to test the proposed hypotheses is challenging, precisely because of this complex relationship, but exogenous factors that affect ASR and marriage rules may offer a way out. For example, to our knowledge there is currently no research that empirically tests whether levels of polygamy dampen the effect of sex ratios on the relative bargaining power of women and men by changing the demand for partners. Future work may address this question by studying gendered bargaining dynamics in a population where the level of polygamy regionally varies owing to ecological factors unrelated to gender ideology. Legislative changes of legal differences between regions can further provide natural experiments for studying how marriage rules or freedom of choice in marriage and divorce can constrain people's ability to gain leverage from a biased sex ratio. More broadly, understanding the causal role that gender norms might play would benefit from more longitudinal work, particularly from areas undergoing rapid cultural and economic change. Alternatively, experimental norm-change studies that specifically posit causal direction in the study design can be another way to see how gender norms might alter beliefs about processes that affect the ASR (e.g. sex-biased investment).

## 5. Conclusion

A growing body of research addresses the effect of sex ratios on reproductive behaviour in humans. This research follows predictions from sexual conflict theory and findings from the non-human animal literature, where sex ratios have been shown to affect mating strategies and bargaining in many species in line with predictions. When a population's sex ratio is biased, this changes both the interests of each sex in parenting and mating strategies, as well as individuals’ bargaining power in sexual conflicts of interest. Furthermore, the costs and benefits of different mating strategies in turn can influence sex ratios, resulting in a dynamic relationship between sex ratios and mating and parenting behaviour. The fitness payoffs of these behaviours are also dependent on environmental factors, such as mortality risks associated with parenting or mate search, which can constrain the effect of sex ratios. In humans, sexual conflict dynamics are even more complex owing to the role of cultural norms regarding gender, and here we believe the term ‘gendered conflict’ is useful in directing researchers’ focus to this additional layer of complexity (Lawson et al., 2023).

In this paper we argue that sexual conflict theory must be revised in order to understand these processes in human populations, where culture adds a layer of complexity that cannot be ignored. Gender norms themselves are in part a product of conflicts of interest between women and men. However as cultural traits they are also subject to cultural evolutionary processes, resulting in their detachment from expected sexual conflict outcomes and their potential to in turn influence conflict dynamics. Gender norms play a central role in marriage market and bargaining dynamics by altering who is on the market, how individuals are valued and how much freedom of choice they have, and by directly affecting sex ratios. Crucially, gender norms may also structure how individuals respond to market value gained or lost through biased sex ratios. Integrating sexual conflict theory and cultural evolution theory is crucial to understanding gendered conflict dynamics.

## Data Availability

n/a

## Research transparency and reproducibility

n/a
